# Type I Interferonopathies in Children: An Overview

**DOI:** 10.3389/fped.2021.631329

**Published:** 2021-03-31

**Authors:** Debora M. d'Angelo, Paola Di Filippo, Luciana Breda, Francesco Chiarelli

**Affiliations:** ^1^Department of Pediatrics, University of Chieti, Chieti, Italy; ^2^Center of Excellence on Aging, University of Chieti, Chieti, Italy

**Keywords:** autoinflammatory disease, type I interferon (IFN) signaling, Aicardi-Goutières syndrome, Janus kinase inhibitors, innate immunity, interferon

## Abstract

Notable advances in gene sequencing methods in recent years have permitted enormous progress in the phenotypic and genotypic characterization of autoinflammatory syndromes. Interferonopathies are a recent group of inherited autoinflammatory diseases, characterized by a dysregulation of the interferon pathway, leading to constitutive upregulation of its activation mechanisms or downregulation of negative regulatory systems. They are clinically heterogeneous, but some peculiar clinical features may lead to suspicion: a familial “idiopathic” juvenile arthritis resistant to conventional treatments, an early necrotizing vasculitis, a non-infectious interstitial lung disease, and a panniculitis associated or not with a lipodystrophy may represent the “interferon alarm bells.” The awareness of this group of diseases represents a challenge for pediatricians because, despite being rare, a differential diagnosis with the most common childhood rheumatological and immunological disorders is mandatory. Furthermore, the characterization of interferonopathy molecular pathogenetic mechanisms is allowing important steps forward in other immune dysregulation diseases, such as systemic lupus erythematosus and inflammatory myositis, implementing the opportunity of a more effective target therapy.

## Background

In the last 20 years, remarkable progress has been made on the phenotypic and genotypic characterization of autoinflammatory disorders (1, 2). The onset of an autoinflammatory phenotype early in infancy is often linked to a monogenic autoinflammatory syndrome, whose diagnosis is currently a challenge for pediatricians (2). Interferonopathies represent the most recent and increasingly featured chapter among these pathologies. The term “interferonopathy” first appeared in 2003, when some authors identified phenotypic overlaps between Aicardi–Goutieres syndrome (AGS) encephalopathy, viral congenital infections, and some autoimmune diseases such as systemic lupus erythematosus (SLE), postulating a common pathological feature as an upregulation of interferon (IFN) α activity (3). Indeed, AGS was originally defined as pseudo-TORCH (toxoplasmosis, rubella, cytomegalovirus, and herpes) syndrome, identifying a group of serologically negative disorders that mimic congenital TORCH infections, suggesting a similar pathogenetic mechanism (3, 4). In the first decade of the 2000s, the genes underlying AGS and monogenic forms of SLE were partially identified and in both cases were associated with type I IFN signaling, confirming the involvement of a common pathway (4–7). In the meantime, the role of nucleic acids and the activation of intracellular receptors for the production of type I IFN by virus-infected cells was identified (4). These considerations led to the definition of a new group of mendelian disorders, characterized by the mutation of single genes involved in the type I IFN signaling: the interferonopathies (8). Further genetic analysis of these patients showed mutations in genes involved in several overlapping pathways: the pathogenesis, still largely to be characterized, lies in alterated response to nucleic acid stimuli or defective regulation of downstream effector molecules (8–10). Despite clinical heterogeneity, some phenotypic characteristics in this group of diseases are shared: early onset of the skin vasculopathy with chilblains, livedo reticularis and panniculitis, involvement of central nervous system (CNS), and interstitial lung disease are the most represented (2, 4, 11). These clinical manifestations represent the “clinical IFN signature.”

## Type I Interferon Signaling: Regulation and Dysregulation

In 1957, Isaacs and Lindenmann described a soluble factor in supernatant of cells incubated with influenza virus, able to “interfere” with cell viral infection *in vitro*; they called this factor “interferon” (12). IFN was subsequently isolated and characterized, showing important biological properties such as inhibition of viral growth and cell multiplication and many immunomodulatory activities (2, 4, 13). Three types of IFNs are described: type I IFN primarily participates in the innate immune system response to viral antigens; type II or IFN-gamma mainly activates Th1 and B lymphocyte adaptive system response; and type III or IFN-lambda has antiviral and modulating properties (13, 14). Precisely, type I IFNs are powerful inflammatory polypeptides, ubiquitously expressed by immune and non-immune cells, including macrophages, lymphocytes, dendritic cells, fibroblasts, and hematopoietic plasmacytoid dendritic cells. This family of cytokines are all induced by microbial and viral nucleic acids, and it consists of the predominantly produced interferon-α (13 variants) and interferon-β and other less frequent variants such as IFN-ε, -κ, -τ, and -ζ (14–20). Indeed, virus infection leads to an intracytoplasmic accumulation of viral double-stranded (ds) RNA or DNA. This surplus of nucleic acids exceeded the activation threshold and is sensed in the cytoplasm or endosomes by different pattern recognition receptors as Toll-like receptors (TLRs), RIG-I-like receptors (RLRs), NOD-like receptors (NLRs), and other cytoplasmic DNA receptors such as the cyclic GMP-AMP synthase (cGAS) (16–21). In particular, intracellular dsDNA interacts with the enzyme cGAS, activating the production of cyclic 2′3′GMP-AMP (cGAMP). In the endoplasmic reticulum (ER), cGAMP binds and activates the STimulator of INterferon Genes (STING), which translocates into the Golgi apparatus. Here, STING C-terminal tail (CTT) recruits TANK-binding kinase 1 (TBK1), which phosphorylates serine 365 of CTT. Phospho-S365 induces the coupling and the subsequent phosphorylation by TBK1 of IFN regulatory factors 3 (IRF3), which translocates to the nucleus and induces the transcription of IFN-β and of IFN regulatory factors 7 (IRF7), which is responsible for IFN-α secretion and autocrine signaling amplification (22).

**Figure 1:**
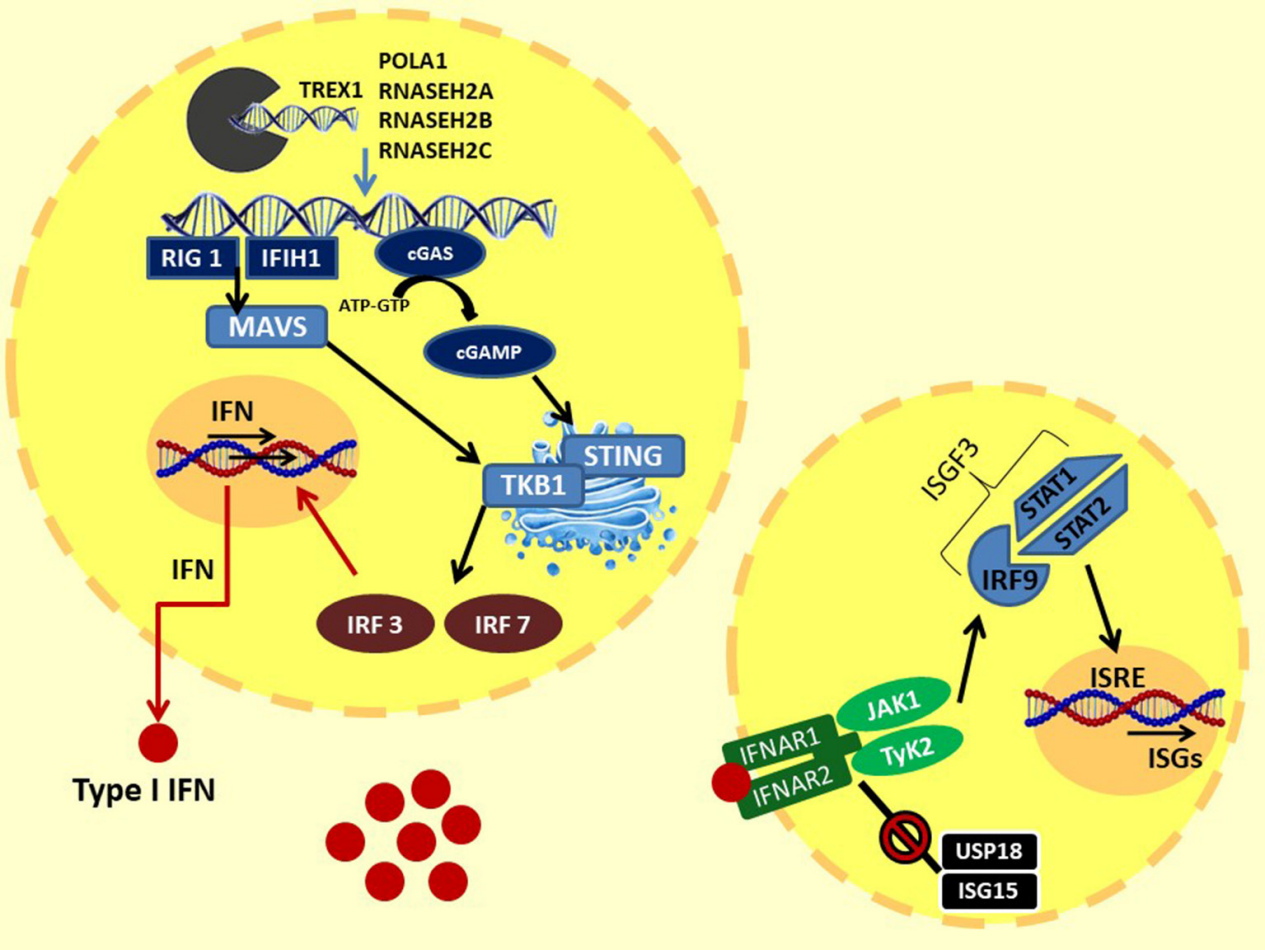
Type I IFN signaling: the intracytoplasmatic accumulation of viral (or endogenous due to loss of function of the enzymes responsible for degradation) nucleic acids is sensed by two systems: (1) the cytoplasmic DNA receptors cyclic GMP-AMP synthase cGAS: intracellular dsDNA activate cGAS, leading to the production of cGAMP. cGAMP binds and activates STING in ER, which translocates into the Golgi apparatus. Here, the STING C-terminal tail recruits TBK1. TBK1 induces the coupling and the phosphorylation of IFR3 and, consequently, IFR7. (2) The cytosolic RNA helicases RIG-I–MDA5 system: intracellular dsRNA activates MDA5 and RIG I, which bind and activate MAVS in the mitochondrial membrane, forming the MAVS signaling complex. MAVS triggers downstream TBK1, than activating IRF3 and IRF7. Finally, IRF3 and IRF7 translocate to the nucleus and induce the transcription of IFN-β and IFN-α, respectively. Type I IFNs, through autocrine and paracrine action, bind to IFN receptors. The IFNR dimerization recruits JAK1 and TyK2 proteins. This activation promotes the STAT1–STAT2 dimerization and the binding of IRF9 to assemble the heterotrimeric transcription complex ISGF3. In the nucleus, ISGF3 binds to IFN-stimulated response elements (ISRE), promoting the expression of interferon-stimulated genes (ISGs). At the same time, IFN signaling is regulated by a negative feedback mechanism by the USP18–ISG15 system. The USP18 binds the IFNAR2 subunit, decoupling it from JAK1 and inhibiting the propagation of the next signal. ISG15 prevents the degradation of UBS18 by SPK2. TREX1, DNA 3′–repair exonuclease 1; RNASEH2, ribonuclease H2; POLA1, polymerase-α; cGAS, GMP-AMP synthase; cGAMP, 2′3′GMP-AMP; STING, STimulator of INterferon Genes; TBK1, TANK-binding kinase 1; MAVS, mitochondrial antiviral-signaling protein; IRF3, IFN regulatory factors 3; IRF7, IFN regulatory factors; RIG-I, retinoic acid-inducible gene-I; MDA5, melanoma differentiation-associated gene 5; JAK1, Janus kinase 1; TyK2, tyrosine kinase 2; IRF9, IFN regulatory factors 9; ISGF3, interferon-stimulated gene factor 3; USP18, ubiquitin-specific peptidase 18; ISG15, interferon-stimulated gene 15; SPK2, S-phase kinase-associated protein 2.

Another pathway for IFN activation includes three cytosolic RNA helicases: the RIG-I-like helicases (*RLHs*), the retinoic acid inducible gene-I (*RIG-I*), and the melanoma differentiation-associated gene 5 (*MDA5*), which recognize viral dsRNA in the cytoplasm. *RIG-I* and *MDA5* activate downstream IKK epsilon and TBK1, which phosphorylate and activate IRF3 and IRF7, allowing IFNα and β gene transcription (23). Therefore, type I IFNs, produced by the infected cells and released in the extracellular environment, perform an autocrine and paracrine action binding to the heterodimeric interferon receptors (IFNRs), which are constituted by the subunits IFN-α receptor 1 (IFNAR1) and IFNAR2. IFN bound to its receptor induces dimerization of these two subunits, which recruit JAK1 (Janus kinase 1) and TyK2 (tyrosine kinase 2) proteins. This activation further promotes the STAT1–STAT2 dimerization and the recruitment of IFN regulatory factors 9 (IRF9) to assemble the heterotrimeric transcription complex interferon-stimulated gene factor 3 (*ISGF3*). In the nucleus, ISGF3 binds to IFN-stimulated response elements (ISRE) promoting the expression of interferon-stimulated genes (*ISGs*) (14, 20, 24). *ISG* expression controls chemotaxis, cell migration, apoptosis, and cell proliferation, and it regulates immune detection and defense against infections (20). Moreover, a negative feedback mechanism finely regulates signal transduction through the IFN receptor: the ubiquitin-specific peptidase 18 (USP18)–interferon-stimulated gene 15 (*ISG15*) system. The USP18 binds the IFNAR2 subunit, decoupling it from JAK1 and inhibiting the propagation of the next signal. These events induce a refractory state in the cells with reduced sensitivity to further stimulation. ISG15 is an IFN-induced protein that extracellularly stimulates the production of type II IFN, and intracellularly it binds lysine residues of proteins (ISGylation); this process can be reversed by USP18. In addition, the unconjugated ISG15 form prevents the degradation of UBS18 by the S-phase kinase-associated protein 2 (SPK2), activating a negative regulation of the IFNR signaling (25, 26). Moreover, IFNR activation stimulates the transcription of the suppressor of cytokine signaling 1 (SOCS1), activating another negative feedback mechanism. Indeed, SOCs 1 binds TyK2, reducing IFN signal transduction and, simultaneously, the surface expression of IFNR1, which is stabilized by Tyk2 (27). The IFN pathway described above is illustrated in Figure 1.

**Figure 2:**
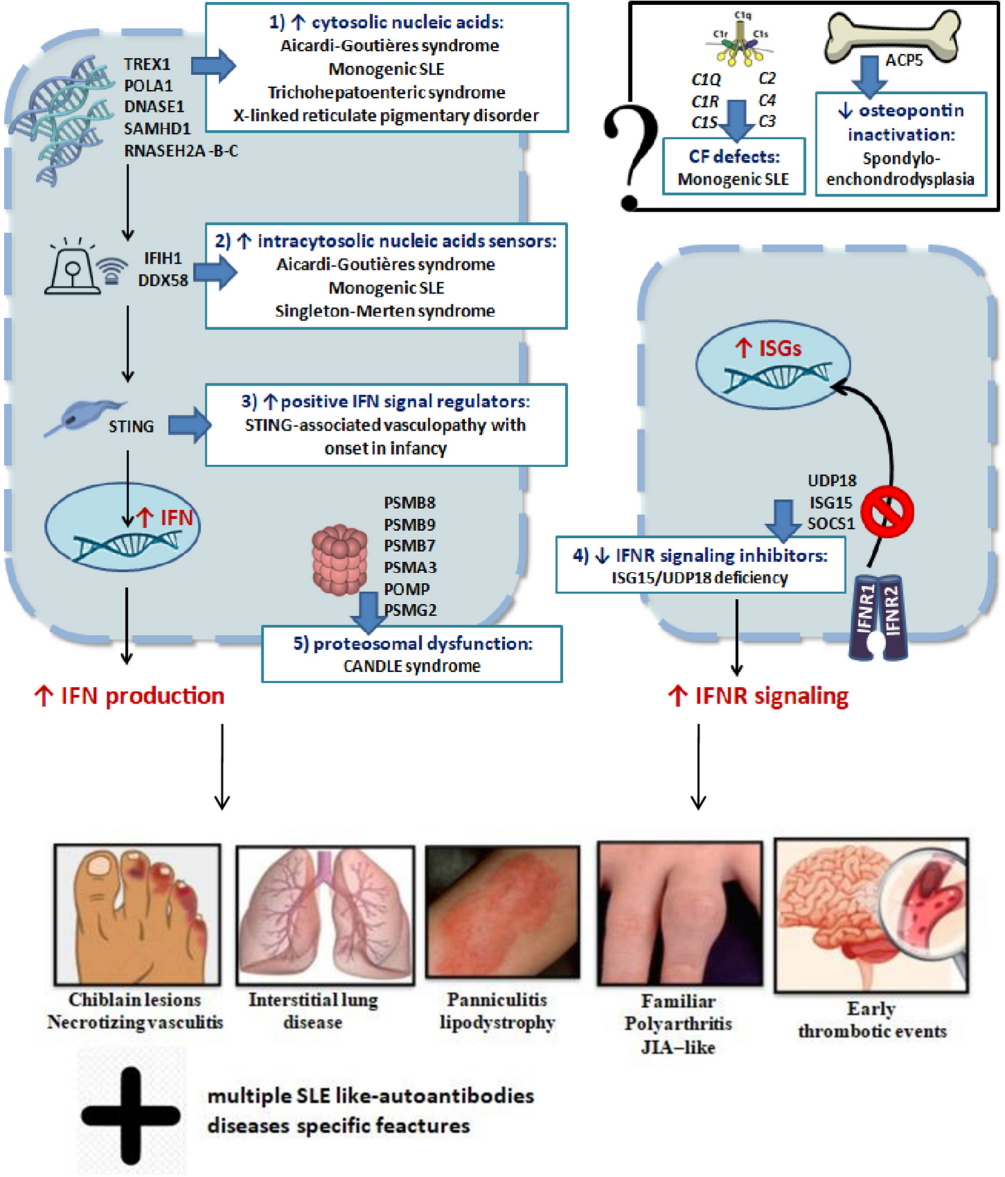
Schematic representation of genetic mechanisms and clinical manifestations in main interferonopathies (see in the text).

In conclusion, dysregulation of type I IFN signaling can occur through at least five mechanisms: (I) cytosolic accumulation of endogenous nucleic acids by loss-of-function mutations of genes that encode for enzymes responsible for DNA or DNA–RNA hybrid molecule degradation; (II) intracytosolic nucleic acid sensor alteration and following activation of the signal below the threshold; (III) gain-of-function mutations of positive IFN signaling regulators, leading to constitutive activation of the IFN pathway; (IV) loss-of-function mutations of negative IFN signaling regulators; (V) proteasomal dysfunction leading to increased IFN signaling through a mechanism being defined (2, 4). The role of mutations of genes encoding osteopontin activity proteins (TRAP/ACP5) and complement factors in interferonopathies is still not yet clarified (2). These mechanisms are summarized in Figure 2. Very recently, biallelic mutations of two components of the histone pre-mRNA processing complex were implicated: the U7 snRNA-associated Sm-like protein (LSM11) and the RNA U7 Small Nuclear 1 (RNU7-1). Such gene alterations are responsible for a modified stoichiometric conformation of the histones and an altered chromatin formation, with dysregulation of the cGAS-STING activity. These findings highlight further pathways of constitutive activation of IFN, which need to be investigated (28). The main genes involved and their role in the pathogenesis of interferonopathies are summarized in Table 1.

**Table 1:** Mutated gene and corresponding locus, protein function, pattern of inheritance, and common symptoms of main type I interferonopathies.

**Disease**	**Gene**	**Locus**	**Protein function**	**Inheritance**	**Phenotype**
AGS 1	*TREX*-*1*	3p21.31	3′-5′ DNA exonuclease	AR /AD	Basal ganglia calcifications, delayed psycho-motor development, epilepsy (classic AGS)
AGS2	*RNASEH2B*	13q14.3	Components of Rnase H2 complex Removes ribonucleotides from RNA-DNA hybrids	AR	Classic AGS
AGS3	*RNASEH2C*	11.q.13.1			Classic ASG
AGS4	*RNASEH2A*	19p13.13			ASG, dysmorfic features
AGS5	*SAMHD1*	20q11.23	Restricts the availability of cytosolic deoxynucleotides	AR	Severe neurological involvement with early stroke, arthropathy
AGS6	*ADAR*	1q21.3	RNA-specific adenosine deaminase 1-dsRNA preventing recognition by MDA5 receptor	AR /AD	ASG, bilateral striatal necrosis
AGS7	*IFIH1*	2q24.3	Cytosolic receptor for dsRNA	AD	Mild AGS
SPENCD	*ACP5*	2q24.2	Negative regulation of Osteopontine	AR	Spondyloenchondrodysplasia, autoimmunity
SAVI	*TMEM173* or *STING*	5q31.1	Transduction of cytoplasmic DNA-induced signal	AD	Skin vasculopaty, bilateral interstitial lung disease
PRAAS	*PSMB8* *PSMB9* *PSMB7* *PSMA3* *POMP* *PSMG2*	6p21.32 6p21.32 9q33.3 14q23.1 13q12.3 18p11.21	Proteasome complex subunit Proteosome chaperone	AR	(CANDLE syndrome) Chronic Neutrophilic Dermatosis panniculitis with Lipodystrophy, Elevated Temperature and myalgia, hepatomegaly, splenomegaly, brain calcifications
ISG15 deficiency	*ISG15*	1p31.33	Stabilizes USP18	AR	Neurological involvement, mycobacterial susceptibility
USP18 deficiency	*UDP18*	2q37.1	Negative regulator of IFNR signaling	AD	Neurological involvement (pseudo TORCH syndrome), hepatomegaly, thrombocytopenia
SMS	*IFIH1*	2q24.3	Cytosolic receptor for dsRNA	AD	Dental and skeletal dysplasia, aortic calcification, glaucome and psoriasis
Atypical SMS	*DDX58*	9p21.1	Cytosolic receptor for dsRNA	AD	SMS without dental dysplasia
THES	*SKIV2L*	6p21.33	RNA helicase	AR	Severe intractable diarrhea, trichorrehexis nodosa, facial dysmorfism, immunodeficit
DNAse II deficiency	*DNAse II*	19p13.13	Endonuclease	AR	Severe neonatal anemia, membranoproliferative glomerulonephritis, liver fibrosis, deforming arthropathy
XLPDR	*POLA1*	Xp22.11-p21.3	DNA Polymerase Alpha 1	X-linked	Generalized hyperpigmentation with small hypomelanotic macules during early childhood, dysmorphic feature, recurrent respiratory infections, and severe gastrointestinal disorders

*AD, autosomal dominant; AR, autosomal recessive; ADAR1, adenosine deaminase acting on RNA 1; ACP5, Acid Phosphatase 5, Tartrate Resistant; AGS, Aicardi-Goutières syndrome; DDX58, DEAD Box Protein 58; IFIH1, IFN-induced helicase C domain-containing protein 1; ISG15, Interferon-stimulated gene 15; PSMB8, Proteasome subunit beta type-8; PSMB9, Proteasome subunit beta type-9; PSMB7, Proteasome subunit beta type-7; PSMA3, Proteasome 20S Subunit Alpha 3; POMP, Proteasome Maturation Protein; PSMG2, Proteasome Assembly Chaperone 2; RNASEH2, Ribonuclease H2; SAMHD1, deoxynucleoside triphosphate triphosphohydrolase SAM domain and HD domain 1; SPENCD, spondyloenchondrodysplasia; SAVI, STING associated vasculopathy with onset in infancy; PRAAS, Proteasome Associated Autoinflammatory Syndromes; SMS, Singleton-Merten syndrome; THES, Trichohepatoenteric syndrome; TMEM173, transmembrane Protein 173; TREX1, DNA 3′-repair exonuclease 1; TRAP, Acid Phosphatase 5 Tartrate Resistant; IFIH1, IFN-induced helicase C domain-containing protein 1; MDA5, melanoma differentiation-associated protein 5; XLPDR, X-linked reticulate pigmentary disorder*.

## Type I IFN and Brain

Neurological involvement is a prominent feature of most interferonopathies, especially with specific mutations (Table 1) (29, 30). Recent studies showed that IFN type I is implicated in regulating microglial function, both during development and in response to neuroinflammation, ischemia, and neurodegeneration (30, 31). The microglia is the local component of the CNS innate immune system, and it plays a central role in the modulation of CNS inflammatory homeostasis. Microglia, indeed, is essential in phagocytizing myelin debris and providing support to oligodendrocytes and axons, also during synaptogenesis. Disruption in microglial activation may result in detrimental effects within white matter (30–32). Therefore, a disruption in the fine balance between required clearance and excessive phagocytosis is the basis of neurodegenerative pathologies. Type I IFN plays a significant role in regulating this balance.

The effects of IFN on CNS and microglia seem to be dual and opposite (31). Experimental murine models on multiple sclerosis (MS), autoimmune encephalomyelitis, and stroke demonstrated that IFNβ (produced by microglia) has local modulatory effects on myelin inflammation, by increasing phagocytosis (including T cells phagocytosis) and promoting the blood–brain barrier integrity. These data suggest a therapeutic role of IFN in these neurodegenerative diseases (33–35, 35, 36). On the other hand, it was demonstrated that a chronic upregulation of type I IFN signaling by microglia may be injurious and this effect may be greater in white matter (31). Dysregulation of the IFN pathway in mouse knockout for the *USP18* gene led to a severe neurological phenotype with seizures, tremor(s), and early mortality (37, 38). Pathological brain evaluation highlighted hydrocephalus with basal ganglia calcifications and clusters of microglia in the white matter which strongly resembled the neuropathological pattern of several human microgliopathies (37, 38). The evaluation of USP18-deficient human patients confirmed the same neuroinflammatory phenotype, with the same astrocyte and microglial activation (25, 39). The susceptibility of nervous tissue to IFN insult seems to be prominent in the fetal CNS, when the process of neurogenesis, synaptogenesis, synaptic pruning, and myelization is paramount (30).

This condition reflects the very early onset of ganglion calcifications, epilepsy, and psychomotor retardation in some interferonopathies, such as AGS (29, 40). Moreover, recent studies suggest that many neuropathies, including Alzheimer's disease and MS, can be considered “microgliopathies,” and the microglia and the IFN signaling pathway may represent a promising therapeutic target (30, 41, 42).

## Main Clinical Syndromes and Associated Molecular Defects

Genetic mutations and clinical syndromes associated with interferonopathies are continuously and rapidly evolving. The first diseases identified as type I interferonopathies were Aicardi–Goutières syndrome (AGS), the spondyloenchondrodysplasia (SPENCD), and the monogenic forms of systemic lupus erythematosus (SLE) (4). According to the 2017 classification of the International Union of Immunological Societies (IUIS), 13 type I interferonopathies were identified. In addition to those initially described, other monogenic syndromes were recognized, including proteasome-associated autoinflammatory syndromes (PRAAS), ISG15 deficiency, Singleton–Merten syndrome (SMS), and STING-associated vasculopathy with onset in infancy (SAVI) (43, 44). Clinical and pathogenetic characteristics of the main interferonopathies are described below.

## Aicardi–Goutières Syndrome

AGS is a progressive encephalopathy with onset within the first year of life, characterized by basal ganglia calcifications, chronic cerebrospinal fluid (CSF) lymphocytosis, and elevated type I IFN levels in the CSF without evidence of any congenital infection (2, 45). The currently recognized genetic mutations associated with AGS are seven (Table 1), and they led to the identification of seven subgroups of the disease (AGS 1–7). The autosomal recessive forms are caused by mutations of genes encoding for intracellular nucleic acids degradation enzymes: 3′-5′ DNA exonuclease -TREX1 (AGS1), ribonucleases as the *RNASEH2B* (AGS2), *RNASEH2C* (AGS3), and *RNASEH2A* (AGS4), SAM and HD domain-containing deoxynucleoside triphosphate triphosphohydrolase 1-*SAMHD1* (AGS5), and adenosine deaminase acting on RNA 1-*ADAR1* (AGS6). The autosomal dominant forms are mainly caused by mutations of genes encoding for the intracellular RNA sensor IFN-induced helicase C domain-containing protein 1-IFIH1 (AGS7) (2, 5, 46–52). A less severe phenotype was reported in patients with *ISG15* gene mutations (51).

AGS is a clinically heterogeneous disorder. Two forms of AGS were described: in the early-onset form, psychomotor development delay and liver abnormalities are evident from birth; in the later-onset AGS, after the first weeks or months of normal development, symptoms appear as a progressive decline in head growth, spasticity, and cognitive and developmental delay that range from moderate to severe. In addition, abnormal eye movements, nystagmus, glaucoma, and poor visual behavior are often associated (5, 47–52); a peculiar characteristic is a “startle reactions,” in response to even minimal sensory stimuli (53). Intracranial calcifications, white matter destruction, and brain atrophy are evident on neuroimaging, similar to radiological findings seen in congenital infections. Epileptic seizures are present in a variable percentage from 10–30% to 53–75%. In children with the late-onset form, as symptoms lessen, clinical manifestations stabilize with no further worsening of the disease (54). Stroke and cerebral aneurysms are common manifestations in patients affected by mutation of SAMHD1 gene (55). Extraneurological symptoms mainly involve the skin (35% of cases): chilblain-like lesions, characterized by areas of inflammation and necrosis secondary to peripheral inflammatory vasculopathy, are localized mainly in fingers and toes or in the auricles, mostly in the cold months. Other common alterations include thrombocytopenia, hepatosplenomegaly, elevated transaminases, psoriasis, interstitial lung disease, and intermittent fever, which erroneously cause a misdiagnosis with acute CNS infections (meningitis, encephalitis) (45, 46, 48, 50). An autoimmune SLE-like habitus associated with type 1 diabetes mellitus, hypothyroidism, polygammaglobulinemia, and hemolytic anemia has been often described (56). Furthermore, arthropathy with progressive contractures were reported only in children with *SAMHD1* gene mutations (57).

## Spondyloenchondrodysplasia With Immune Dysregulation

SPENCD is a rare autosomic recessive disorder characterized by multiple skeletal dysplasia, neurological involvement, and immune dysfunction with an increased IFN type I signature in peripheral blood and urine, due to mutations of tartrate-resistant acid phosphatase (*TRAP*) gene (ACP5) (58, 59). TRAP enzyme plays a role in inhibiting the activity of osteopontin (Opn), a protein involved in bone metabolism and immune system function modulation. Opn is expressed by osteoclasts and osteoblasts, and it stimulates bone remodeling and turnover. In addition, it has been shown that secreted Opn acts as a “cytokine” that polarizes lymphocytic differentiation in Th1, promoting macrophage production of IL12. *ACP5* gene mutations impair or eliminate TRAP ability to inactivate Opn (58). The role of Opn in modulating type I IFN production is still being defined. Some studies showed that intracellular expression of Opn in plasmacytoid dendritic cells was required for TLR9-dependent expression of IFN-α and colocalization of Opn was essential for efficient nuclear translocation of IRF7 and associated IFNα gene expression. In addition, Opn-deficient mice developed impaired IFNα-dependent natural killer cell responses to tumors and reduced IFN-α responses after infection with herpes simplex virus 1 (HSV-1) (60). Consequently, bone dysplasias with enchondromatosis, platyspondyly and irregularity of vertebral endplates and short stature, brain calcifications with spasticity and autoimmune disorders, including SLE with malar rash, anti-phospholipid syndrome, and glomerulonephritis are the main clinical manifestations (58, 60, 61). Sjögren's syndrome, hemolytic anemia, thrombocytopenia, hypothyroidism, inflammatory myositis, Raynaud's disease, and vitiligo were also described (58, 59).

## Monogenic Systemic Lupus Erythematosus

Increased IFNα serum level in SLE patients was widely demonstrated (62–66). Moreover, increased damage or death of cells and defective nucleic acid metabolism and the consequent production of autoantibodies against nucleic acids are characteristic of SLE. Accordingly, accumulation of nucleic acids may activate the same pathways involved in interferonopathies with an overproduction of IFN, suggesting an overlap between type I interferonopathies and SLE (67, 68). Recently, childhood-onset SLE was linked to single-gene mutations, defining monogenic or familiar SLE. This evolving monogenic mutations can be grouped into four classes: (I) complement factors, (II) enzymes involved in the endogenous metabolism of nucleic acids (extracellular DNASE), (III) proteins directly involved in IFN type I pathway, and (IV) factors involved in the regulation of B and T lymphocyte self-tolerance (69–71). Main genes involved in these forms are summarized in Table 2.

**Table 2:** The main altered genes described in the monogenic forms of SLE and corresponding phenotype.

**Locus**	**Gene location**	**Protein**	**Inheritance**	**Pathway**	**Phenotype**
*C1QA* *C1QB* *C1QC*	1p36.12	C1q	AR	Complement—Classic pathway	Nephritis, CNS involvement, photosensibility, arthritis, infectious susceptibility
*C1R*	12p13.31	C1r	AR	Complement—Classic pathway	Poliarthicular arthritis, upper and lower respiratory tract infections
*C1S*	12p13.31	C1s	AR	Complement—Classic pathway	Hashimoto's thyroiditis, autoimmune epatitis
*C2*	6p21.33	C2	AR	Complement—Classic pathway	Arthritis, malar rash, discoid rash, and photosensibility. Rare neurological and renal involvement
*C4A-B*	6p21.33	C4	AR	Complement—Classic pathway	Multiorgan involvement with severe nephritis and photosensibility
*C3*	6p21.33	C4	AR	Complement—Classic and no classic pathway	Complement deficiencies with upper and lower respiratory tract infection, SLE in a minority of affected individuals
*DNASE 1*	16p13.3	DNASE1	AD	Extracellular acid nucleic degradation	Sjogren syndrome, high level of antinucleosomal autoantibodies
*DNASE1L3*	3p14.3	DNASE1L3	AR	Extracellular acid nucleic degradation	Early onset, nephritis, ANCA positive hypocomplementemic urticarial vasculitis syndrome (HUVS)
*ACP5*	2q24.2	TRAP	AD	Type-I IFN—regulation of Opn	Cytopenia (also implied in SMS)
*IFIHI*	2q24.3	MDA-5	AD	Type-I IFN Cytosolic sensor for dsRNA	IgA deficiency, mild lower limb Spasticity (also implied in SMS and AGS)
*TREX1*	3p21.31	TREX1	AD	Type-I IFN degradation of intracellular ds-ss DNA	FCL (also implied in AGS)
*SAMHD1*	20q11.23	SAMHD1	AR	Type I IFN cytoplasmic ssRNA/DNA sensor	FCL (also implied in AGS)
*PRKCD*	3p21.2	PRKCD	AR	Self-tolerance-B-cell survival and proliferation	Renal involvement, CNS vasculitis, lymphoproliferative syndromes
*RAG2*	11p12	RAG2	AR/AD	Self-tolerance	Immune-mediated cytopenias to localized destructive vasculitis
*TNFRSF6*	10q23.31	CD95	AR	Self-tolerance- apoptosis	Marked lymphadenopathy

*AD, autosomal dominant; AR, autosomal recessive; ACP5-TRAP, Acid Phosphatase 5 Tartrate Resistant; IFIH1, IFN-induced helicase C domain-containing protein 1; MDA5, melanoma differentiation-associated protein 5; TREX1, DNA 3′ repair exonuclease; SAMHD1, deoxynucleoside triphosphate triphosphohydrolase SAM domain and HD domain 1; PRKCD, Protein Kinase C Delta; RAG 2, recombination-activating genes 2; TNFRSF6, Truncated tumor necrosis factor receptor superfamily member 6; FLC, familial chilblain lupus*.

Complement protein defects are responsible for autosomal recessive forms of juvenile onset-SLE. C1q deficiency is the strongest risk factor known for the development of SLE, followed by C4A defects. The link between complement protein deficiency and IFN type I upregulation is currently under investigation. A defect of the opsonization of apoptotic self-bodies and, therefore, of the cellular clearance and the “efferocytosis” is considered the main causative factor (69). In addition, it was demonstrated that C1q *in vitro* inhibits peripheral blood mononuclear cell (PBMCs) and plasmacytoid dendritic cell IFNα production, thus hypothesizing a direct C1q regulatory role (72). The clinical phenotype is represented by an early onset of SLE with frequent early renal glomerular involvement and increased susceptibility to pyogenic infections (especially upper and lower tract respiratory infections) (2, 69). In addition, complement 1r subcomponent (C1r) mutations were described, with a clinical phenotype characterized by prominent neurological involvement and lymphadenopathy (73).

The role of endonuclease gene defects in the development of early SLE was demonstrated. In the early 2000s, some authors highlighted that reduced serum endonuclease DNASE1 activity was linked to the development of autoantibodies (especially anti-nucleosomal autoantibodies) and active SLE disease in patients and mice (74–76). The deficiency of DNASE1L3, a homolog of DNASE1, was first described in 2011 as a pediatric-onset SLE correlated with lupus nephritis (77). Recently, it was demonstrated that DNASE1L3 acts extracellularly and it plays a primary role in digesting genomic DNA circulating microparticles derived from apoptotic cells. Indeed, these DNA microparticles were targeted by autoantibodies in the serum of DNASE1L3-deficient mice and patients and the treatment with exogenous DNASE1L3 inhibited antibody binding. These findings suggest a central role of DNASI1L3 in immune tolerance regulation and in autoantibodies development in SLE (78, 79). Furthermore, the role of another endonuclease, DNAse 2, was recently described. Biallelic mutations in DNASE2 gene were associated with a clinical picture ranging from membrano-proliferative glomerulonephritis and liver fibrosis to severe neonatal anemia (80).

*TREX1* is a genetic locus encoding for a form of 3′-5′ DNA exonuclease associated with monogenic SLE (81–85). Single-nucleotide polymorphisms (SNPs) in *TREX1* were found associated with common forms of SLE (83–85). Early-onset familial chilblain lupus (FCL) is due to an autosomal dominant mutation of *TREX1*. It is a rare form of cutaneous SLE characterized by cold-induced severe vasculitic ulcerative lesions of (fingers, toes) and ears (81, 82). In addition, mutations in *TREX1* with autosomal dominant inheritance were related to retinal vasculopathy with cerebral leukodystrophy (RVCL). Type I IFN signature was described in these patients. RVCL is characterized by middle-age onset of progressive visual loss due to retinal vasculopathy (telangiectasias, microaneurysms, retinal macular vascular obliteration) and neurologic manifestations including hemiparesis, facial paralysis, aphasia, and hemianopsia up to psychiatric disorders (86). Besides, a homozygous variant of this gene was identified in patients with an early-onset cerebral SLE (87). Also, mutations in *SAMHD1* have been reported in patients affected by FCL, with and without vascular CNS involvement (88). Among monogenic SLE associated with mutations of genes involved in the IFN signal regulation, in addition to *ISG15* and *USP18* gene (discussed in other sections), it is important to mention the role of OTU domain-containing protein 1 (OTUD1). *OTUD1* gene encodes for a deubiquitinase that interacts with IRF3, removing the poly-ubiquitin chains on IRF3 and suppressing IFN gene transcription. Patients with loss-of-function missense mutations in *OTUD1* present with many different autoimmune disease, including early onset-SLE (71).

## Proteasome-Associated Autoinflammatory Syndromes

PRAAS are a heterogeneous group of interferonopathies caused by inherited loss-of-function mutations in genes encoding proteasome subunits of 20S core particles such as PSMB8, PSMB9, PSMB7, and PSMA3 or proteasome chaperone factors such as POMP and PSMG2 (Table 1). These mutations lead to a failure of proteasome complex formation, causing an alteration of intracellular protein homeostasis and the accumulation of ubiquitinated proteins (2, 89–92). The underlying molecular mechanisms that link this alteration to the activation of the IFN pathway are not yet fully understood. Recently, new insights came from the unfolded protein response (UPR), which originated in response to an accumulation of misfolded proteins in the ER (ER stress). Indeed, defective proteasome compromises the ER-associated protein degradation, resulting in an accumulation of misfolded ER proteins in the lumen. This alteration is determined by three intra ER membrane-resident proteins, in particular the inositol-requiring enzyme 1α/β (IRE1), with subsequent activation of a signal pathway that allows NF-κB activation and IRF3 factor transcription, leading to a IFN-depending inflammation (92, 93).

PRAAS include the Japanese autoinflammatory syndrome with lipodystrophy (JASL) and chronic atypical neutrophilic dermatosis with lipodystrophy and elevated temperature syndrome (CANDLE) syndromes (2). Common clinical features are the presence of pernio-like purplish nodular lesions (neutrophilic dermatosis), panniculitis with progressive lipodystrophy and muscle atrophy, and joint contractures with extremity deformity. Hepatosplenomegaly and hypochromic or hemolytic anemia were also reported. An early metabolic syndrome, with systemic hypertension and dyslipidemia, occurs in 40–80% of patients (89–92). Proteasome defects were also associated with neurologic diseases. In murine models, loss of proteosome subunits led to development of microcephaly and aberrant rostrocaudal patterning of the branchial arches, suggesting a role of some proteasome components in neuronal development (89–93). Indeed, a mild cognitive delay was also described in some CANDLE/PRAAS patients (89).

## ISG15 Deficiency

ISG15 deficiency consists of two clinical overlapping forms: an immunodeficiency phenotype and a type I interferonopathy (2, 94). It was first described in two families from Turkey and Iran as a Mendelian susceptibility to mycobacterial disease (MSMD) (94). Indeed, these mutations alter the leukocyte secretion of ISG15. Inducing IFNγ, ISG15 plays an essential role in optimizing the anti-mycobacterial immune response by Th1 lymphocytes and macrophages. It also downregulates the IFNR signaling pathway by stabilizing USP18 (2, 14). An upregulation of type IFN I, mostly characterized by a neurological phenotype with basal ganglia calcifications and epilepsy, was described (2, 4). Recently, loss-of-function mutations of ISG15 and signs of IFN activation in keratinocytes, skin endothelial cells, and peripheral blood myeloid cells were reported in five patients affected by skin ulcerative lesions in the perineal, groin, scalp, neck, and axillary areas. These findings may suggest another phenotype with a unique pattern of skin localization of vasculopathic inflammation (95).

## Singleton–Merten Syndrome

In 1973, Singleton et al. described a syndrome characterized by dental dysplasia (periodontitis, root resorption), thoracic and aortic calcifications, osteoporosis, and skeletal radiographic changes (distal limb osteolysis, widened medullary cavities) in two children (96). Later, an atypical form of SMS was identified in two families and it was characterized by abnormal valvular and thoracic calcifications and osteoporosis without dental alterations (97). In addition, psoriatic-like lesions and glaucoma are other clinical features described in SMS. Genetic and pathogenetic bases of this disease were identified about 40 years later. The SMS phenotype results in an upregulation of IFN type I due to gain-of-function mutations of the genes *IFIH1* (typical form) and *DDX58* (atypical form), encoding for 2 intracytoplasmic nucleic acid sensors (*MDM2* and *RIG-1*, respectively), activating the IFN pathway (97–99).

## Sting-Associated Vasculopathy With Onset IN Infancy

SAVI was first described in 2014 in six children affected by systemic inflammation, severe cutaneous vasculopathy, and early interstitial lung disease. In these patients, heterozygous gain-of-function mutations in the transmembrane protein 173 (*TMEM173*) gene result in an upregulation of the IFN gene transcription (100).

Skin and lungs are certainly the most involved systems. Telangiectatic lesions on nose and cheek and violaceous atrophic plaques and nodules on hands are typical clinical signs. Capillary tortuosity with subsequent loss of distal capillary vascularity is responsible for painful ulcerative lesions (especially on fingers and toes, ears, and nose). These lesions evolve into eschars, up to digital amputation, ear cartilage reabsorption, perforation of the nasal septum, or dystrophic nail changes. Skin biopsy revealed capillary wall inflammation with fibrin deposits in the lumen and a dense neutrophilic infiltrate with karyorrhexis throughout the vessel wall, in the absence of granulomatous lesions. Interstitial lung disease with fibrosis together with hilar or paratracheal lymphadenopathy is the main responsible of morbidity and mortality (101). Patients experience a progressive hypoxemic respiratory insufficiency, often asymptomatic in early childhood. Lung biopsy highlighted mixed lymphocytic inflammatory infiltrate (100, 101).

Autoantibodies ANA and cANCA were found in some of these patients, resulting in a difficult differential diagnosis with childhood granulomatosis and polyangiitis (102). Failure to thrive, intermittent fever, polyarthritis, and myositis are other clinical features described in SAVI. Recent reports identified a phenotype mimicking the rheumatoid factor-positive polyarticular juvenile idiopathic arthritis (JIA) associated with an interstitial lung disease (103).

## Trichohepatoenteric Syndrome

Trichohepatoenteric syndrome (THES) is a very rare disease characterized by typical wooly, brittle, and patchy hair (trichorrhexis nodosa), neonatal-onset intractable diarrhea, facial dysmorphism (“coarse” face feature), and intrauterine growth restriction with short stature. Additional findings include poorly characterized immunodeficiency with recurrent infections and liver disease. Mild intellectual disability was reported in about 50% of affected individuals. Less common findings include congenital heart defects and platelet anomalies.

The disease is caused by loss-of-function mutations of tetratricopeptide repeat domain 37 gene (*TTC37*) and Ski2-like RNA helicase gene (*SKIV2L*), which encode for two homologous proteins participating in human RNA exosome homeostasis, with consequent dysregulation of altered mRNA turnover. Moreover, SKIV2L seems to modulate directly RIG-I-like receptors (104).

## X-Linked Reticulate Pigmentary Disorder

XLPDR is a rare genodermatosis presenting as an early onset reticulated skin hyperpigmentation associated with recurrent pneumonia and enterocolitis, resembling inflammatory bowel disease. A distinctive face characterized by an upswept frontal hairline and arched eyebrows, severe photophobia with corneal inflammation, and failure to thrive were also described.

XLPDR is caused by an intronic mutation that disrupts the expression of POLA1, which encodes the catalytic subunit of DNA polymerase-α (POLA1). It was highlighted that POLA1 is required for synthesis of RNA–DNA hybrids, which negatively modulate the activation of the IFN regulatory factors (105). Since this is an intronic mutation, it can only be detected by whole genome sequencing (not exone sequencing) (44, 105).

## COPA Syndrome

The genetic analysis of patients suffering from a familial form of juvenile “idiopathic” arthritis associated with hemorrhagic interstitial lung disease permitted to identify a new autoinflammatory disease: the COPA syndrome. Indeed, heterozygous mutations were detected in the gene coding for the alpha subunit of the coatomer complex (COPa), which plays a role in the intracytosolic proteins retro-transport between Golgi apparatus-ER. An immune dysregulation of Th17 cells associated with inflammatory cytokine upregulation was described, probably triggered by intracellular ER-stress (106).

A role of IFN signal dysregulation in the pathogenesis was suspected because of the common clinical and pathogenetic features of SAVI syndrome and the finding of IFN-I pathway activation in peripheral blood of COPA syndrome patients. However, the inclusion of COPA syndrome among interferonopathies is still to be defined (44).

## Approach To The Diagnosis of Interferonopathies

For a pediatrician, the first step of the diagnostic workup of these new hereditary diseases is their awareness and knowledge. This is a group of autoinflammatory pathologies that are very different from the more known “inflammasomopathies;” indeed, the typical clinical features are not periodic fever, nor are urticarious skin rash or recurrent serositis. Conversely, an early necrotizing vasculitis, a non-infectious interstitial lung disease in the context of an inflammatory clinical picture, a panniculitis associated with or without lipodystrophy, and early thrombotic events are peculiar elements that should lead to the suspicion of an interferonopathy (2, 44). Type I interferonopathies should also be considered when an autoimmune disease presents a very early onset and/or it is refractory to standard treatment and/or it occurs in several members of the same family (2). For example, a clinical and serological SLE-like syndrome in early life (particularly prepubertal age) should lead to the exclusion of a monogenic form (2). Clinical and laboratory “red flags” for interferonopathies are summarized in Table 3.

**Table 3:** Red flags of interferonopathies.

**Clinical red flags in pediatric settings**	**Laboratory red flags**
*Neonatal setting:* • TORCH-like syndrome without infection • Encephalopathy with skin involvement	• Systemic inflammation with leukopenia • Increased ESR with normal or slightly increased CRP • Fluctuating low-titer ANA and other autoantibodies • Leukocytes and IFN increased in cerebrospinal fluid • IFN signature
*Neurological setting:* • Leukodystrophy, especially if associated with fever, chilblains, and/or cytopenia • Spasticity, demyelination, seizures, microcephaly associated to skin manifestations and/or glaucoma • Subacute encephalopathy with basal ganglia calcifications in the first years of life, especially if associated with recurrent fever and microcephaly	
*Rheumatological setting:* • Chiblains • Raynaud's phenomenon • Panniculitis/lipodystrophy • Recurrent unexplained fever • Inflammation signs with slightly increased CRP • Autoimmune characteristics similar to SLE • Poor efficacy of common biologic drug used in other autoinflammatory diseases	
*Pulmonary setting:* • Interstitial lung disease associated with skin manifestations like painful ulcerative lesions evolving into digital amputation • Pulmonary arterial hypertension	

*CRP, C reactive protein; SLE, Systemic Lupus Erythematosus; ESR, Erythrocyte Sedimentation Rate; ANA, Antinuclear antibodies; IFN, Interferon*.

In case of suspicion, IFN signaling should always be evaluated. Type I IFN protein dosage is not available in routine clinical practice because of very low circulating levels. However, it was demonstrated that type I IFN pathway upregulation was correlated with increased expression of a subset of six ISG (IFI27, IFI44L, IFIT1, ISG15, RSAD2, and SIGLEC1) by quantitative polymerase chain reaction assays with whole blood samples in AGS patients, the so-called *interferon signature*. So, they developed the “interferon score” with a high sensitivity for AGS, which may be used both for diagnosis and follow-up and to assess disease activity (107). The IFN signature seems to be also very sensitive to differentiate monogenic and complex type I interferonopathies from inflammasomopathies. However, standardization between different centers could be difficult (44). Therefore, new “interferon scores” were proposed for autoimmune diseases such as SLE or anti-phospholipid antibody syndrome (ALS) using NanoString technology and new standardization methods are being studied to make the reporting homogeneous in research laboratories (108–110).

Since interferonopathies are hereditary genetic diseases, the conclusive diagnosis is the genetic sequencing, with the identification of causative mutations (2, 44).

However, in clinical practice undifferentiated systemic autoinflammatory diseases (USAIDs) are very frequent. More recently, some authors developed a standardized screening for “IFN signature,” using the type-I IFN-response-gene score (IRG-S), cytokine profiling, clinical phenotyping, and genetic evaluation by next-generation sequencing (NGS) (whole-exome sequencing or Sanger sequencing) for diagnosis of new or known interferonopathies, in patients with USAIDs (111). The characterization of the genes and the inflammatory pattern in patients with USAIDs, through modern NGS approaches up to exome or whole-genome sequencing, is currently an important goal, in order to identify the potential appropriate therapeutic targets (1, 2, 44).

## Current Therapeutic Options

Type I interferonopathies are often resistant to conventional immunosuppressive treatment. Intravenous boluses of methylprednisolone or immunoglobulins were used in many cases in the acute phase of the disease, without a conclusive relapse control (2, 44, 112).

A very promising therapeutic strategy is represented by JAK inhibitors. Baricitinib and ruxolitinib are selective JAK1/JAK2 inhibitors; tofacitinib inhibits JAK1/JAK3. Treatment *in vitro* of peripheral blood mononuclear cells from a patient with SAVI with tofacitinib, ruxolitinib, or baricitinib resulted in an inhibition of constitutive STAT1 activation (100). Later, the clinical efficacy of JAK1 blockade was confirmed by the improvement of vasculitic lesions and lung function in three children suffering from SAVI, treated with ruxolitinib, and in a 9-year-old boy with a SAVI vasculitis treated with tofacitinib (113, 114). The suppression of type I IFN activation during treatment with ruxolitinib was also shown in two children with AGS (115). Recently, the efficacy of baricitinib in the treatment of pediatric interferonopathies was demonstrated: 18 patients were enrolled, 10 with CANDLE, four with SAVI, and four with genetically undefined pathology. Patients treated for a mean of 2.3 years have shown a statistically significant improvement in clinical items and a reduction in the need for glucocorticoids. Additionally, 13 patients had growth potential improved, with catch-up growth observed in nine patients. Bone mineral density also improved, mainly due to steroid-sparing-dependent effects (116). In a study on pharmacokinetics in pediatric and adult patients, a dose-dependent effect of inhibition of the IFN signaling pathway was clearly shown (117). Future studies may clarify other aspects like the efficacy of baricitinib on the neurological involvement (2, 44).

Antiretroviral therapy (RT therapy) usually used for HIV infection is another therapeutic option. In murine studies, *TREX-1* (mutated in AGS) plays a role in the degradation of the reverse-transcribed DNA (cDNA) of the HIV virus. Retroelements and mobile genetic elements represent up to half of the human genome; they can move within the genome by a reverse transcription of an RNA intermediate (cDNA) and insertion of its cDNA, a process called retrotransposition (44, 118). Therefore, the human cDNA may be the main substrate of TREX and the cDNA accumulation could be an important cause of IFN pathway activation. Moreover, recent studies have shown that SAMHD1 and ADAR also act on cDNA, confirming this hypothesis. It has been shown that *TREX1* knockout mice have a reduction in cardiac inflammation by treatment with a combination of the non-nucleoside RT inhibitor nevirapine together with the nucleoside RT inhibitors tenofovir and emtricitabine (118). The intracellular accumulation of retrotransposomes was also demonstrated, using TREX1 deficiency reprogrammed neuronal cells (an experimental model of the AGS); RT inhibitors stavudine and lamivudine reduced the neurotoxicity of AGS neurons and organoids (119). Recently, the results of a single-center, open-label pilot study involving 11 patients with AGS treated with abacavir, lamivudine, and zidovudine for 12 months were published, demonstrating a reduction in IFN signature and IFN activity in the cerebrospinal fluid and an increase in cerebral blood flow in three of the five patients evaluated. These data support the possible role of HIV RT inhibitors in the treatment of interferonopathies (120).

Another desirable option for treatment of type I interferonopathies are monoclonal antibodies targeting IFN-α (sifalimumab) and IFNAR (anifrolumab). A multicenter, phase 2, open-label study was completed for sifalimumab in patients with SLE, showing good tolerability (121). A recent study confirmed the efficacy against placebo of anifrolumab in the control of skin and joint lesions of 362 SLE patients, showing a significant reduction in the IFN signature of these patients (122). These data highlight their potential use in therapeutic regimens in interferonopathies. Another currently theoretical strategy may be to modulate IFNAR degradation, through regulation of its ubiquitination by histone deacetylase 11 (HDH11) (123).

Finally, future possibility may be allogenic stem cell transplantation and autologous stem cell transplantation after gene correction of a patient's mutation (44).

## Conclusions

Type I interferonopathies represent an emerging group of autoinflammatory disorders, with clinical characteristics and therapeutic response very different from previously characterized “inflammasome diseases.” Pediatricians must be aware of the clinical “IFN alarm” signals (such as chilblain-like vasculitis and interstitial lung disease) in order to suspect them and to direct properly the diagnostic workup with eminent prognostic implications. Molecular and biochemical details of IFN signaling permitted the identification of the etiopathological substrate of these rare syndromes, implementing the possibility of a target therapy. Nowadays, thanks to new sequencing techniques such as NGS, many indefinite autoinflammatory syndromes can often assume a genetic and clinical “identity,” offering these children the hope of new therapeutic “tailored” and “customized” options.

## Author Contributions

All authors contributed equally to this manuscript and approved the final version and its submission to the journal.

## Conflict of Interest

The authors declare that the research was conducted in the absence of any commercial or financial relationships that could be construed as a potential conflict of interest.

## Glossary

AGS, Aicardi–Goutieres syndrome; SLE, systemic lupus erythematosus; IFN, interferon; toxoplasmosis, rubella, cytomegalovirus, and herpes, TORCH; CNS, central nervous system; TLRs, Toll-like receptors; RLRs, RIG-I-like receptors; NLRs, NOD-like receptors; cGAS, cyclic GMP-AMP synthase; cGAMP, 2′3′GMP-AMP; STING, STimulator of INterferon Genes; ER, endoplasmic reticulum; TBK1, TANK-binding kinase 1; IRF3, IFN regulatory factors 3; IRF7, IFN regulatory factors 7; RLHs, RIG-I-like helicases; RIG-I, retinoic acid-inducible gene-I; MDA5, melanoma differentiation-associated gene 5; IFNRs, interferon receptors; IRF9, IFN regulatory factors 9; ISGs, interferon-stimulated genes; USP18, ubiquitin-specific peptidase 18; ISG15, interferon-stimulated gene 15; SPK2, S-phase kinase-associated protein 2; AD, autosomal dominant; AR, autosomal recessive; ADAR1, adenosine deaminase acting on RNA 1; ACP5, acid phosphatase 5, tartrate resistant; AGS, Aicardi–Goutières syndrome; DDX58, DEAD Box Protein 58; IFIH1, IFN-induced helicase C domain-containing protein 1; ISG15, interferon-stimulated gene 15, PSMB8 (proteasome subunit beta type-8; PSMB9, proteasome subunit beta type-9; PSMB7, proteasome subunit beta type-7; PSMA3, proteasome 20S subunit alpha 3; POMP, proteasome maturation protein; PSMG2, proteasome assembly chaperone 2; RNASEH2, ribonuclease H2; SAMHD1, deoxynucleoside triphosphate triphosphohydrolase SAM domain and HD domain 1; SPENCD, spondyloenchondrodysplasia; SAVI, STING-associated vasculopathy with onset in infancy; PRAAS, proteasome-associated autoinflammatory syndromes; SMS, Singleton–Merten syndrome; THES, trichohepatoenteric syndrome; TMEM173, transmembrane protein 173; TREX1, DNA 3′—repair exonuclease 1; TRAP, acid phosphatase 5 tartrate resistant; IFIH1, IFN-induced helicase C domain-containing protein 1; XLPDR, X-linked reticulate pigmentary disorder; MS, multiple sclerosis; CSF, cerebrospinal fluid; Opn, osteopontin; HSV-1, herpes simplex virus 1; PBMCs, peripheral blood mononuclear cells; SNPs, single-nucleotide polymorphisms; UPR, unfolded protein response; IRE1, inositol requiring enzyme 1α/β; TTC37, tetratricopeptide repeat domain 37 gene; SKIV2L, Ski2-like RNA helicase gene; ALS, anti-phospholipid antibody syndrome; USAIDs, undifferentiated systemic autoinflammatory diseases; NGS, next-generation sequencing; SOCS1, suppressor of cytokine signaling 1; LSM11, U7 snRNA-associated Sm-like protein; RNU7-1, RNA U7 small nuclear 1; OTUD1, OTU domain-containing protein 1; C1r, complement 1r subcomponent.

## References

[1] Papa R Picco P Gattorno M. The expanding pathways of autoinflammation: a lesson from the first 100 genes related to autoinflammatory manifestations. Adv Protein Chem Struct Biol. (2020) 120:1–44. 10.1016/bs.apcsb.2019.11.001 32085880

[2] Volpi S Picco P Caorsi R Candotti F Gattorno M. Type I interferonopathies in pediatric rheumatology. Rheumatology. (2016) 14:35. 10.1186/s12969-016-0094-4 27260006 PMC4893274

[3] Crow YJ Black DN Ali M Bond J Jackson AP Lefson M. Cree encephalitis is allelic with Aicardi-Goutières syndrome: implications for the pathogenesis of disorders of interferon alpha metabolism. J Med Genet. (2003) 40:183–7. 10.1136/jmg.40.3.183 12624136 PMC1735395

[4] Rodero MP Crow YJ. Type I interferon-mediated monogenic autoinflammation: the type I interferonopathies, a conceptual overview. J Exp Med. (2016) 213:2527–38. 10.1084/jem.20161596 27821552 PMC5110029

[5] Rice GI Bond J Asipu A Brunette RL Manfield IW Carr IM. Mutations involved in Aicardi-Goutières syndrome implicate SAMHD1 as regulator of the innate immune response. Nat Genet. (2009) 41:829–32. 10.1038/ng.373 19525956 PMC4154505

[6] Crow YJ Hayward BE Parmar R Robins P Leitch A Ali M. Mutations in the gene encoding the 3'-5' DNA exonuclease TREX1 cause Aicardi-Goutières syndrome at the AGS1 locus. Nat Genet. (2006) 38:917–20. 10.1038/ng1845 16845398

[7] Elkon KB Stone VV. Type I interferon and systemic lupus erythematosus. J Interferon Cytokine Res. (2011) 31:803–12. 10.1089/jir.2011.0045 21859344 PMC3216059

[8] Crow YJ. Type I interferonopathies: a novel set of inborn errors of immunity. Ann N Y Acad Sci. (2011) 1238:91–8. 10.1111/j.1749-6632.2011.06220.x 22129056

[9] Eleftheriou D Brogan PA. Genetic interferonopathies: an overview. Best Pract Res Clin Rheumatol. (2017) 31:441–59. 10.1016/j.berh.2017.12.002 29773266

[10] Crow YJ. Type I interferonopathies: mendelian type I interferon up-regulation. Curr Opin Immunol. (2015) 32:7–12. 10.1016/j.coi.2014.10.005 25463593

[11] Davidson S Steiner A Harapas CR Masters SL. An update on autoinflammatory diseases: interferonopathies. Curr Rheumatol Rep. (2018) 20:38. 10.1007/s11926-018-0748-y 29846818

[12] Isaacs A Lindenmann J. Virus interference. I. The interferon. Proc R Soc Lond B Biol Sci. (1957) 147:258–67. 10.1098/rspb.1957.0048 26297790

[13] Negishi H Taniguchi T Yanai H. The interferon (IFN) class of cytokines and the IFN regulatory factor (IRF) transcription factor family. Cold Spring Harb Perspect Biol. (2018) 10:a028423. 10.1101/cshperspect.a028423 28963109 PMC6211389

[14] Trinchieri G. Type I interferon: friend or foe? J Exp Med. (2010) 207:2053–63. 10.1084/jem.20101664 20837696 PMC2947062

[15] Gough DJ Messina NL Clarke CJP Johnstone RW Levy DE. Constitutive type I interferon modulates homeostatic balance through tonic signaling. Immunity. (2012) 36:166–74. 10.1016/j.immuni.2012.01.011 22365663 PMC3294371

[16] Ivashkiv LB Donlin LT. Regulation of type I interferon responses. Nat Rev Immunol. (2014) 14:36–49. 10.1038/nri3581 24362405 PMC4084561

[17] Schroder K Hertzog PJ Ravasi T Hume DA. Interferon-gamma: an overview of signals, mechanisms and functions. J Leukoc Biol. (2004) 75:163–89. 10.1189/jlb.0603252 14525967

[18] Uddin S Platanias LC. Mechanisms of type-I interferon signal transduction. J Biochem Mol Biol. (2004) 37:635–41. 10.5483/BMBRep.2004.37.6.635 15607020

[19] Guiducci C Coffman RL Barrat FJ. Signalling pathways leading to IFN-alpha production in human plasmacytoid dendritic cell and the possible use of agonists or antagonists of TLR7 and TLR9 in clinical indications. J Intern Med. (2009) 265:43–57. 10.1111/j.1365-2796.2008.02050.x 19093959

[20] Schneider WM Chevillotte MD Rice CM. Interferon-stimulated genes: a complex web of host defenses. Ann Rev Immunol. (2014) 32:513–45. 10.1146/annurev-immunol-032713-120231 24555472 PMC4313732

[21] Li T Chen ZJ. The cGAS–cGAMP–STING pathway connects DNA damage to inflammation, senescence, and cancer. J Exp Med. (2018) 215:1287–99. 10.1084/jem.20180139 29622565 PMC5940270

[22] Dobbs N Burnaevskiy N Chen D Gonugunta VK Alto NM Yan N. STING activation by translocation from the ER is associated with infection and autoinflammatory disease. Cell Host Microbe. (2015) 18:157–68. 10.1016/j.chom.2015.07.001 26235147 PMC4537353

[23] Reikine S Nguyen JB Modis Y. Pattern recognition and signaling mechanisms of RIG-I and MDA5. Front Immunol. (2014) 5:342. 10.3389/fimmu.2014.00342 25101084 PMC4107945

[24] Michalska A Blaszczyk K Wesoly J Bluyssen HAR. A positive feedback amplifier circuit that regulates interferon (IFN)-stimulated gene expression and controls type I and type II IFN responses. Front Immunol. (2018) 28:1135. 10.3389/fimmu.2018.01135 29892288 PMC5985295

[25] Zhang X Bogunovic D Payelle-Brogard B Francois-Newton V Speer SD Yuan C. Human intracellular ISG15 prevents interferon-alpha/beta over-amplification and auto-inflammation. Nature. (2015) 517:89–93. 10.1038/nature13801 25307056 PMC4303590

[26] Ketscher L Knobeloch KP. ISG15 uncut: dissecting enzymatic and non-enzymatic functions of USP18 *in vivo*. Cytokine. (2015) 76:569–71. 10.1016/j.cyto.2015.03.006 25805508

[27] Schreiber G. The molecular basis for differential type I interferon signaling. J Biol Chem. (2017) 292:7285–94. 10.1074/jbc.R116.774562 28289098 PMC5418031

[28] Uggenti C Lepelley A Depp M Badrock AP Rodero MP El-Daher MT. cGAS-mediated induction of type I interferon due to inborn errors of histone pre-mRNA processing. Nat Genet. (2020) 52:1364–72. 10.1038/s41588-020-00737-3 33230297

[29] Goldmann T Blank T Prinz M. Fine-tuning of type I IFN-signaling in microglia-implications for homeostasis, CNS autoimmunity and interferonopathies. Curr Opin Neurobiol. (2016) 36:38–42. 10.1016/j.conb.2015.09.003 26397019 PMC7126514

[30] McDonough A Lee RV Weinstein JR. Microglial interferon signaling and white matter. Neurochem Res. (2017) 42:2625–38. 10.1007/s11064-017-2307-8 28540600 PMC5777301

[31] Hosmane S Tegenge MA Rajbhandari L Uapinyoying P Kumar NG Thakor N. Toll/interleukin-1 receptor domain-containing adapter inducing interferon-beta mediates microglial phagocytosis of degenerating axons. J Neurosci. (2012) 32:7745–57. 10.1523/JNEUROSCI.0203-12.2012 22649252 PMC3398425

[32] Anderson SR Vetter ML. Developmental roles of microglia: a window into mechanisms of disease. Dev Dyn. (2019) 248:98–117. 10.1002/dvdy.1 30444278 PMC6328295

[33] Prinz M Schmidt H Mildner A Knobeloch KP Hanisch UK Raasch J. Distinct and nonredundant *in vivo* functions of IFNAR on myeloid cells limit autoimmunity in the central nervous system. Immunity. (2008) 28:675–86. 10.1016/j.immuni.2008.03.011 18424188

[34] Veldhuis WB Derksen JW Floris S Van Der Meide PH De Vries HE Schepers J. Interferon-beta blocks infiltration of inflammatory cells and reduces infarct volume after ischemic stroke in the rat. J Cereb Blood Flow Metab. (2003) 23:1029–39. 10.1097/01.WCB.0000080703.47016.B6 12973019

[35] Inacio AR Liu Y Clausen BH Svensson M Kucharz K Yang Y. Endogenous IFN-beta signaling exerts anti-inflammatory actions in experimentally induced focal cerebral ischemia. J Neuroinflammation. (2015) 12:211. 10.1186/s12974-015-0427-0 26581581 PMC4652356

[36] Yang C Hawkins KE Doré S Candelario-Jalil E. Neuroinflammatory mechanisms of blood-brain barrier damage in ischemic stroke. Am J Physiol Cell Physiol. (2019) 316:C135–53. 10.1152/ajpcell.00136.2018 30379577 PMC6397344

[37] Knobeloch KP Utermohlen O Kisser A Prinz M Horak I. Reexamination of the role of ubiquitin-like modifier ISG15 in the phenotype of UBP43-deficient mice. Mol Cell Biol. (2005) 25:11030–4. 10.1128/MCB.25.24.11030-11034.2005 16314524 PMC1316970

[38] Schwabenland M Mossad O Peres AG Kessler F Maron FJM Harsan LA. Loss of USP18 in microglia induces white matter pathology. Acta Neuropathol Commun. (2019) 7:106. 10.1186/s40478-019-0757-8 31272490 PMC6610953

[39] Meuwissen ME Schot R Buta S Oudesluijs G Tinschert S Speer SD. Human USP18 deficiency underlies type 1 interferonopathy leading to severe pseudo-TORCH syndrome. J Exp Med. (2016) 213:1163–74. 10.1084/jem.20151529 27325888 PMC4925017

[40] Crow YJ Zaki MS Abdel-Hamid MS Abdel-Salam G BoespflugTanguy O Cordeiro NJV. Mutations in ADAR1, IFIH1, and RNASEH2B presenting as spastic paraplegia. Neuropediatrics. (2014) 45:386–91. 10.1055/s-0034-1389161 25243380

[41] Feng X Bao R Li L Deisenhammer F Arnason BGW Reder AT. Interferon-β corrects massive gene dysregulation in multiple sclerosis: short-term and long-term effects on immune regulation and neuroprotection. EBioMedicine. (2019) 49:269–83. 10.1016/j.ebiom.2019.09.059 31648992 PMC6945282

[42] Choubey D. Type I interferon (IFN)-inducible Absent in Melanoma 2 proteins in neuroinflammation: implications for Alzheimer's disease. J Neuroinflammation. (2019) 16:236. 10.1186/s12974-019-1639-5 31771614 PMC6880379

[43] Picard C Bobby Gaspar H Al-Herz W Bousfiha A Casanova JL Chatila T. International union of immunological societies: 2017 primary immunodeficiency diseases committee report on inborn errors of immunity. J Clin Immunol. (2018) 38:96–128. 10.1007/s10875-017-0464-9 29226302 PMC5742601

[44] Yu ZX Song HM. Toward a better understanding of type I interferonopathies: a brief summary, update and beyond. World J Pediatr. (2020) 16:44–51. 10.1007/s12519-019-00273-z 31377974

[45] Crow YJ Manel N. Aicardi-Goutières syndrome and the type I interferonopathies. Nat Rev Immunol. (2015) 15:429–40. 10.1038/nri3850 26052098

[46] Crow YJ Chase DS Lowenstein Schmidt J Szynkiewicz M Forte GMA Gornall HL. Characterization of human disease phenotypes associated with mutations in TREX1, RNASEH2A, RNASEH2B, RNASEH2C, SAM HD1, ADAR, and IFIH1. Am J Med Genet. (2015) 167:296–312. 10.1055/s-0036-1592307 PMC4382202 25604658

[47] Rice GI Forte GMA Szynkiewicz M Chase DS Aeby A Abdel-Hamid MS. Assessment of interferon-related biomarkers in Aicardi-Goutières syndrome associated with mutations in TREX1, RNASEH2A, RNASEH2B, RNASEH2C, SAMHD1, and ADAR: a case-control study. Lancet Neurol. (2013) 12:1159–69. 10.1016/S1474-4422(13)70258-8 24183309 PMC4349523

[48] Zheng S Lee PY Wang J Wang S Huang Q Huang Y. Interstitial lung disease and psoriasis in a child with Aicardi-Goutières syndrome. Front Immunol. (2020) 11:985. 10.3389/fimmu.2020.00985 32508843 PMC7251162

[49] Cuadrado E Michailidou I van Bodegraven EJ Jansen MH Sluijs JA Geerts D. Phenotypic variation in Aicardi-Goutières syndrome explained by cell-specific IFN-stimulated gene response and cytokine release. J Immunol. (2015) 194:3623–33. 10.4049/jimmunol.1401334 25769924

[50] Crow YJ Rehwinkel J. Aicardi-Goutieres syndrome and related phenotypes: linking nucleic acid metabolism with autoimmunity. Hum Mol Genet. (2009) 18:R130–6. 10.1093/hmg/ddp293 19808788 PMC2758706

[51] Coquel F Silva MJ Técher H Zadorozhny K Sharma S Nieminuszczy J. SAMHD1 acts at stalled replication forks to prevent interferon induction. Nature. (2018) 557:57–61. 10.1038/s41586-018-0050-1 29670289

[52] Rice G Patrick T Parmar R Taylor CF Aeby A Aicardi J. Clinical and molecular phenotype of Aicardi-Goutieres syndrome. Am J Hum Genet. (2007) 81:713–25. 10.1086/521373 17846997 PMC2227922

[53] Sun W Manohar S Jayaram A Kumaraguru A Fu Q Li J Allman B. Early age conductive hearing loss causes audiogenic seizure and hyperacusis behavior. Hear Res. (2011) 282:178–83. 10.1016/j.heares.2011.08.004 21872651 PMC3230688

[54] Ramantani G Maillard LG Bast T Husain RA Niggemann P Kohlhase J. Epilepsy in Aicardi-Goutières syndrome. Eur J Paediatr Neurol. (2014) 18:30–7. 10.1016/j.ejpn.2013.07.005 24011626

[55] Thiele H du Moulin M Barczyk K George C Schwindt W Nürnberg G. Cerebral arterial stenoses and stroke: novel features of Aicardi-Goutières syndrome caused by the Arg164X mutation in SAMHD1 are associated with altered cytokine expression. Hum Mutat. (2010) 31:1836–50. 10.1002/humu.21357 20842748 PMC3049152

[56] Cattalini M Galli J Andreoli L Olivieri I Ariaudo G Fredi M. Exploring autoimmunity in a cohort of children with genetically confirmed Aicardi-Goutières syndrome. J Clin Immunol. (2016) 36:693–9. 10.1007/s10875-016-0325-y 27539236

[57] Dale RC Gornall H Singh-Grewal D Alcausin M Rice GI Crow YJ. Familial Aicardi-Goutières syndrome due to SAMHD1 mutations is associated with chronic arthropathy and contractures. Am J Med Genet A. (2010) 152:938–42. 10.1002/ajmg.a.33359 20358604

[58] Briggs TA Rice GI Daly S Urquhart J Gornall H Bader-Meunier B. Tartrate-resistant acid phosphatase deficiency causes a bone dysplasia with autoimmunity and a type I interferon expression signature. Nat Genet. (2011) 43:127–31. 10.1038/ng.748 21217755 PMC3030921

[59] Lausch E Janecke A Bros M Trojandt S Alanay Y De Laet C. Genetic deficiency of tartrate-resistant acid phosphatase associated with skeletal dysplasia, cerebral calcifications and autoimmunity. Nat Genet. (2011) 43:132–7. 10.1038/ng.749 21217752

[60] Shinohara ML Lu L Bu J Werneck MB Kobayashi KS Glimcher LH. Osteopontin expression is essential for interferon-alpha production by plasmacytoid dendritic cells. Nat Immunol. (2006) 7:498–506. 10.1038/ni1327 16604075 PMC3725256

[61] Bilginer Y Düzova A Topaloglu R Batu ED Boduroglu K Güçer S. Three cases of spondyloenchondrodysplasia (SPENCD) with systemic lupus erythematosus: a case series and review of the literature. Lupus. (2016) 25:760–5. 10.1177/0961203316629000 26854080

[62] Kim T Kanayama Y Negoro N Okamura M Takeda T Inoue T. Serum levels of interferons in patients with systemic lupus erythematosus. Clin Exp Immunol. (1987) 70:562–9. 2449306 PMC1542177

[63] Garcia-Romo GS Caielli S Vega B Connolly J Allantaz F Xu Z. Netting neutrophils are major inducers of type I IFN production in pediatric systemic lupus erythematosus. Sci Transl Med. (2011) 3:73ra20. 10.1126/scitranslmed.3001201 21389264 PMC3143837

[64] Kirou KA Lee C George S Louca K Peterson MG Crow MK. Activation of the interferon-alpha pathway identifies a subgroup of systemic lupus erythematosus patients with distinct serologic features and active disease. Arthritis Rheum. (2005) 52:1491–503. 10.1002/art.21031 15880830

[65] Bennett L Palucka AK Arce E Cantrell V Borvak J Banchereau J. Interferon and granulopoiesis signatures in systemic lupus erythematosus blood. J Exp Med. (2003) 197:711–23. 10.1084/jem.20021553 12642603 PMC2193846

[66] Niewold TB Hua J Lehman TJ Harley JB Crow MK. High serum IFN-alpha activity is a heritable risk factor for systemic lupus erythematosus. Genes Immun. (2007) 8:492–502. 10.1038/sj.gene.6364408 17581626 PMC2702174

[67] Moulton VR Suarez-Fueyo A Meidan E Li H Mizui M Tsokos GC. Pathogenesis of human systemic lupus erythematosus: a cellular perspective. Trends Mol Med. (2017) 23:615–35. 10.1016/j.molmed.2017.05.006 28623084 PMC5650102

[68] Ghodke-Puranik Y Niewold TB. Immunogenetics of systemic lupus erythematosus: a comprehensive review. J Autoimmun. (2015) 64:125–36. 10.1016/j.jaut.2015.08.004 26324017 PMC4628859

[69] Omarjee O Picard C Frachette C Moreews M Rieux-Laucat F Soulas-Sprauel P. Monogenic lupus: dissecting heterogeneity. Autoimmun Rev. (2019) 18:102361. 10.1016/j.autrev.2019.102361 31401343

[70] Alperin JM Ortiz-Fernández L Sawalha AH. Monogenic lupus: a developing paradigm of disease. Front Immunol. (2018) 9:2496. 10.3389/fimmu.2018.02496 30459768 PMC6232876

[71] Demirkaya E Sahin S Romano M Zhou Q Aksentijevich I. New horizons in the genetic etiology of systemic lupus erythematosus and lupus-like disease: monogenic lupus and beyond. J Clin Med. (2020) 5:712. 10.3390/jcm9030712 32151092 PMC7141186

[72] Lood C Gullstrand B Truedsson L Olin AI Alm GV Rönnblom L. C1q inhibits immune complex-induced interferon-alpha production in plasmacytoid dendritic cells: a novel link between C1q deficiency and systemic lupus erythematosus pathogenesis. Arthritis Rheum. (2009) 60:3081–90. 10.1002/art.24852 19790049

[73] Demirkaya E Zhou Q Smith CK Ombrello MJ Deuitch N Tsai WL. Brief report: deficiency of complement 1r subcomponent in early-onset systemic lupus erythematosus: the role of disease-modifying alleles in a monogenic disease. Arthritis Rheumatol. (2017) 69:1832–9. 10.1002/art.40158 28544690 PMC5609811

[74] Napirei M Karsunky H Zevnik B Stephan H Mannherz HG Möröy T. Features of systemic lupus erythematosus in Dnase1-deficient mice. Nat Genet. (2000) 25:177–81. 10.1038/76032 10835632

[75] Yasutomo K Horiuchi T Kagami S Tsukamoto H Hashimura C Urushihara M. Mutation of DNASE1 in people with systemic lupus erythematosus. Nat Genet. (2001) 28:313–4. 10.1038/91070 11479590

[76] Martínez Valle F Balada E Ordi-Ros J Vilardell-Tarres M. DNase 1 and systemic lupus erythematosus. Autoimmun Rev. (2008) 7:359–63. 10.1016/j.autrev.2008.02.002 18486922

[77] Al-Mayouf S Sunker A Abdwani R Abrawi SA Almurshedi F Alhashmi N. Loss-of-function variant in DNASE1L3 causes a familial form of systemic lupus erythematosus. Nat Genet. (2011) 43:1186–8. 10.1038/ng.975 22019780

[78] Sisirak V Sally B D'Agati V Martinez-Ortiz W Özçakar ZB David J. Digestion of chromatin in apoptotic cell microparticles prevents autoimmunity. Cell. (2016) 166:88–101. 10.1016/j.cell.2016.05.034 27293190 PMC5030815

[79] Wang L Mi W Li Q-Z. Targeting the extracellular scavenger DNASE1L3 on SLE. J Xiangya Med. (2017) 2:29. 10.21037/jxym.2017.03.03

[80] Rodero MP Tesser A Bartok E Rice GI Della Mina E Depp M. Type I interferon-mediated autoinflammation due to DNase II deficiency. Nat Commun. (2017) 8:2176. 10.1038/s41467-017-01932-3 29259162 PMC5736616

[81] Lee-Kirsch MA Gong M Schulz H Ruschendorf F Stein A Pfeiffer C. Familial chilblain lupus, a monogenic form of cutaneous lupus erythematosus, maps to chromosome 3p. Am J Hum Genet. (2006) 79:731–7. 10.1086/507848 16960810 PMC1592563

[82] Rice G Newman WG Dean J Patrick T Parmar R Flintoff K. Heterozygous mutations in TREX1 cause familial chilblain lupus and dominant Aicardi- Goutieres syndrome. Am J Hum Genet. (2007) 80:811–5. 10.1086/513443 17357087 PMC1852703

[83] Rice GI Rodero MP Crow YJ. Human disease phenotypes associated with mutations in TREX1. J Clin Immunol. (2015) 35:235–43. 10.1007/s10875-015-0147-3 25731743

[84] Namjou B Kothari PH Kelly JA Glenn SB Ojwang JO Adler A. Evaluation of the TREX1 gene in a large multi-ancestral lupus cohort. Genes Immun. (2011) 12:270–9. 10.1038/gene.2010.73 21270825 PMC3107387

[85] Hiraki LT Silverman ED. Genomics of systemic lupus erythematosus: insights gained by studying monogenic young-onset systemic lupus erythematosus. Rheum Dis Clin North Am. (2017) 43:415–34. 10.1016/j.rdc.2017.04.005 28711143

[86] Schuh E Ertl-Wagner B Lohse P Wolf W Mann JF Lee-Kirsch MA. Multiple sclerosis-like lesions and type I interferon signature in a patient with RVCL. Neurol Neuroimmunol Neuroinflamm. (2015) 2:e55. 10.1212/NXI.0000000000000055 25566545 PMC4277301

[87] Ellyard JI Jerjen R Martin JL Lee AY Field MA Jiang SH. Identification of a pathogenic variant in TREX1 in early-onset cerebral systemic lupus erythematosus by Whole-exome sequencing. Arthritis Rheumatol. (2014) 66:3382–6. 10.1002/art.38824 25138095

[88] Ravenscroft JC Suri M Rice GI Szynkiewicz M Crow YJ. Autosomal dominant inheritance of a heterozygous mutation in SAMHD1 causing familial chilblain lupus. Am J Med Genet A. (2011) 155A:235–7. 10.1002/ajmg.a.33778 21204240

[89] de Jesus AA Brehm A VanTries R Pillet P Parentelli A-S Montealegre Sanchez GA. Novel proteasome assembly chaperone mutations in PSMG2/PAC2 cause the autoinflammatory interferonopathy CANDLE/PRAAS4. J Allergy Clin Immunol. (2019) 143:1939–43. 10.1016/j.jaci.2018.12.1012 30664889 PMC6565382

[90] Torrelo A Patel S Colmenero I Gurbindo D Lendinez F Hernandez A. Chronic atypical neutrophilic dermatosis with lipodystrophy and elevated temperature (CANDLE) syndrome. J Am Acad Dermatol. (2010) 62:489–95. 10.1016/j.jaad.2009.04.046 20159315

[91] Garg A Hernandez MD Sousa AB Subramanyam L Martinez de Villarreal L dos Santos HG. An autosomal recessive syndrome of joint contractures, muscular atrophy, microcytic anemia, and panniculitis-associated lipodystrophy. J Clin Endocrinol Metab. (2010) 95:58–63. 10.1210/jc.2010-0488 20534754 PMC2936059

[92] Ebstein F Harlowe MCP Studencka-Turski M Kruger E. Contribution of the unfolded protein response (UPR) to the pathogenesis of proteasome-associated autoinflammatory syndromes (PRAAS). Front Immunol. (2019) 10:2756. 10.3389/fimmu.2019.02756 31827472 PMC6890838

[93] Küry S Besnard T Ebstein F Khan TN Gambin T Douglas J. *De novo* disruption of the proteasome regulatory subunit PSMD12 causes a syndromic neurodevelopmental disorder. Am J Hum Genet. (2017) 100:352–63. 10.1016/j.ajhg.2017.01.003 28132691 PMC5294671

[94] Bogunovic D Byun M Durfee LA Abhyankar A Sanal O Mansouri D. Mycobacterial disease and impaired IFN-gamma immunity in humans with inherited ISG15 deficiency. Science. (2012) 337:1684–8. 10.1126/science.1224026 22859821 PMC3507439

[95] Fernandez MM Garcia-Morato MB Gruber C Murias Loza S Malik MNH Alsohime F. Systemic type I IFN inflammation in human ISG15 deficiency leads to necrotizing skin lesions. Cell Rep. (2020) 31:107633. 10.1016/j.celrep.2020.107633 32402279 PMC7331931

[96] Singleton EB Merten DF. An unusual syndrome of widened medullary cavities of the metacarpals and phalanges, aortic calcification and abnormal dentition. Pediatr Radiol. (1973) 1:2–7. 10.1007/BF00972817 4272099

[97] Jang MA Kim EK Now H Nguyen NT Kim WJ Yoo JY. Mutations in DDX58, which encodes RIG-I, cause atypical Singleton-Merten syndrome. Am J Hum Genet. (2015) 96:266–74. 10.1016/j.ajhg.2014.11.019 25620203 PMC4320253

[98] Rutsch F MacDougall M Lu C Buers I Mamaeva O Nitschke Y. A specific IFIH1 gain-of-function mutation causes Singleton-Merten syndrome. Am J Hum Genet. (2015) 96:275–82. 10.1016/j.ajhg.2014.12.014 25620204 PMC4320263

[99] Pettersson M Bergendal B Norderyd J Nilsson D Anderlid BM Nordgren A. Further evidence for specific IFIH1 mutation as a cause of Singleton-Merten syndrome with phenotypic heterogeneity. Am J Med Genet A. (2017) 173:1396–9. 10.1002/ajmg.a.38214 28319323

[100] Liu Y Jesus AA Marrero B Yang D Ramsey SE Sanchez GAM. Activated STING in a vascular and pulmonary syndrome. N Engl J Med. (2014) 371:507–18. 10.1056/NEJMoa1312625 25029335 PMC4174543

[101] Ozen S Sag E. Childhood vasculitis. Rheumatology. (2020) 59:iii95–100. 10.1093/rheumatology/kez599 32348513

[102] Munoz J Rodiere M Jeremiah N Rieux-Laucat F Oojageer A Rice GI. Stimulator of interferon genes-associated vasculopathy with onset in infancy: a mimic of childhood granulomatosis with polyangiitis. JAMA Dermatol. (2015) 151:872–7. 10.1001/jamadermatol.2015.0251 25992765

[103] Clarke SLN Robertson L Rice GI Seabra L Hilliard TN Crow YJ. Type 1 interferonopathy presenting as juvenile idiopathic arthritis with interstitial lung disease: report of a new phenotype. Pediatr Rheumatol Online J. (2020) 18:37. 10.1186/s12969-020-00425-w 32398023 PMC7218611

[104] Eckard SC Rice GI Fabre A Badens C Gray EE Hartley JL. The SKIV2L RNA exosome limits activation of the RIG-Ilike receptors. Nat Immunol. (2014) 15:839–45. 10.1038/ni.2948 25064072 PMC4139417

[105] Starokadomskyy P Gemelli T Rios JJ Xing C Wang RC Li H. DNA polymerase-alpha regulates the activation of type I interferons through cytosolic RNA:DNA synthesis. Nat Immunol. (2016) 17:495–504. 10.1038/ni.3409 27019227 PMC4836962

[106] Vece TJ Watkin LB Nicholas S Canter D Braun MC Guillerman RP. Copa syndrome: a novel autosomal dominant immune dysregulatory disease. J Clin Immunol. (2016) 36:377–87. 10.1007/s10875-016-0271-8 27048656 PMC4842120

[107] Rice GI Melki I Frémond ML Briggs TA Rodero MP Kitabayashi N. Assessment of type I interferon signaling in pediatric inflammatory disease. J Clin Immunol. (2017) 37:123–32. 10.1007/s10875-016-0359-1 27943079 PMC5325846

[108] El-Sherbiny YM Psarras A Yusof MYM Hensor EMA Tooze R Doody G. A novel two-score system for interferon status segregates autoimmune diseases and correlates with clinical features. Sci Rep. (2018) 8: 5793. 10.1038/s41598-018-33062-1 29643425 PMC5895784

[109] Kim H de Jesus AA Brooks SR Liu Y Huang Y VanTries R. Development of a validated interferon score using NanoString Technology. J Interferon Cytokine Res. (2018) 38:171–85. 10.1089/jir.2017.0127 29638206 PMC5963606

[110] Pin A Monasta L Taddio A Piscianz E Tommasini A Tesser A. An easy and reliable strategy for making type I interferon signature analysis comparable among research centers. Diagnostics. (2019) 9:113. 10.3390/diagnostics9030113 31487897 PMC6787630

[111] de Jesus AA Hou Y Brooks S Malle L Biancotto A Huang Y. Distinct interferon signatures and cytokine patterns define additional systemic autoinflammatory diseases. J Clin Invest. (2020) 130:1669–82. 10.1172/JCI129301 31874111 PMC7108905

[112] Bienias M Brück N Griep C Wolf C Kretschmer S Kind B. Therapeutic approaches to type I interferonopathies. Curr Rheumatol Rep. (2018) 20:32. 10.1007/s11926-018-0743-3 29679241

[113] Frémond M-L Rodero MP Jeremiah N Belot A Jeziorski E Duffy D. Efficacy of the Janus kinase 1/2 inhibitor ruxolitinib in the treatment of vasculopathy associated with TMEM173-activating mutations in 3 children. J Allergy Clin Immunol. (2016) 138:1752–5. 10.26226/morressier.57bc1755d462b80290b4d6b9 27554814

[114] Seo J Kang J-A Suh DI Park E-B Lee C-R Choi SA. Tofacitinib relieves symptoms of stimulator of interferon genes (STING)-associated vasculopathy with onset in infancy caused by 2 *de novo* variants in TMEM173. J Allergy Clin Immunol. (2017) 139:1396–9. 10.1016/j.jaci.2016.10.030 28041677

[115] Meesilpavikkai K Dik WA Schrijver B van Helden-Meeuwsen CG Versnel MA van Hagen PM. Efficacy of baricitinib in the treatment of chilblains associated with Aicardi-Goutières syndrome, a type I interferonopathy. Arthritis Rheumatol. (2019) 71:829–31. 10.1002/art.40805 30666809 PMC6593964

[116] Sanchez GAM Reinhardt A Ramsey S Wittkowski H Hashkes PJ Berkun Y. JAK1/2 inhibition with baricitinib in the treatment of autoinflammatory interferonopathies. J Clin Invest. (2018) 128:3041–52. 10.1172/JCI98814 29649002 PMC6026004

[117] Kim H Brooks KM Tang CC Wakim P Blake M Brooks SR. Pharmacokinetics, pharmacodynamics, and proposed dosing of the oral JAK1 and JAK2 inhibitor baricitinib in pediatric and young adult CANDLE and SAVI patients. Clin Pharmacol Ther. (2017) 104:364–73. 10.1002/cpt.936 29134648 PMC6089664

[118] Beck-Engeser GB Eilat D Wabl M. An autoimmune disease prevented by anti-retroviral drugs. Retrovirology. (2011) 8:91. 10.1186/1742-4690-8-91 22067273 PMC3264515

[119] Thomas CA Tejwani L Trujillo CA Negraes PD Herai RH Mesci P. Modeling of TREX1-dependent autoimmune disease using human stem cells highlights L1 accumulation as a source of neuroinflammation. Cell Stem Cell. (2017) 21:319–31. 10.1016/j.stem.2017.07.009 28803918 PMC5591075

[120] Rice GI Meyzer C Bouazza N Hully M Boddaert N Semeraro M. Reverse-transcriptase inhibitors in the Aicardi-Goutières syndrome. N Engl J Med. (2018) 379:2275–7. 10.1056/NEJMc1810983 30566312

[121] Takeuchi T Tanaka Y Matsumura R Saito K Yoshimura M Amano K. Safety and tolerability of sifalimumab, an anti-interferon-α monoclonal antibody, in Japanese patients with systemic lupus erythematosus: a multicenter, phase 2, open-label study. Mod Rheumatol. (2020) 30:93–100. 10.1080/14397595.2019.1583832 30791804

[122] Morand EF Furie R Tanaka Y Bruce IN Askanase AD Richez C. Trial of anifrolumab in active systemic lupus erythematosus. N Engl J Med. (2020) 382:211–21. 10.1056/NEJMoa1912196 31851795

[123] Cao J Sun L Aramsangtienchai P Spiegelman NA Zhang X Huang W. HDAC11 regulates type I interferon signaling through defatty-acylation of SHMT2. Proc Natl Acad Sci USA. (2019) 116:5487–92. 10.1073/pnas.1815365116 30819897 PMC6431144

